# Transcranial Direct Current Stimulation for Depression in Transgender Patient

**DOI:** 10.7759/cureus.38476

**Published:** 2023-05-03

**Authors:** Anne Sauvaget, Samuel Bulteau, Edith Omon, Eleonore Ghazi, Adélaïde Prévotel, Andrew Laurin

**Affiliations:** 1 Faculté de Médecine, Nantes Université, Nantes, FRA; 2 Faculté de Médecine, Nantes Université, Université de Tours, Nantes, FRA; 3 General Practice, Nantes Université, Nantes, FRA; 4 Psychiatry, Centre Hospitalier Universitaire de Nantes, Nantes, FRA; 5 Faculté de Médecine, Nantes Université, Centre Hospitalier Universitaire de Nantes, Nantes, FRA

**Keywords:** psychiatry, neuromodulation, transcranial direct current stimulation, depression, gender-diverse, transgender

## Abstract

Transgender and gender-diverse (TGD) individuals experience higher rates of mood and anxiety disorders than the general public. Transcranial direct current stimulation (tDCS) is an effective and well-tolerated treatment for major depressive disorder, in cisgender patients. With the very recent exception of electroconvulsive therapy (ECT), neuromodulation in TGD patients is not addressed in the literature. We described here the efficacy and tolerability of tDCS in a 22-year-old Caucasian female-to-male transgender individual (hereafter referred to as a male) suffering from a severe non-treatment-resistant major depressive disorder. This case report suggests that combined tDCS and antidepressant therapy have the potential to treat depressive disorders in patients of all gender identities.

## Introduction

Transgender and gender-diverse (TGD) individuals experience an incongruity between the sex they were assigned at birth and their current gender identity. Due to a variety of factors, including stigma and gender minority stress, TGD people experience higher rates of mood and anxiety disorders than the general public [1].

Transcranial direct current stimulation (tDCS) is an effective and well-tolerated treatment for major depressive disorder in cisgender patients when combined with selective serotonin reuptake inhibitors [2]. 

To our knowledge, the literature has never considered the efficacy and tolerability of this brain stimulation procedure in patients with other gender identities; we do so here, with reference to a TGD patient.

## Case presentation

The patient in question, who consented to the publication of this case report, is a 22-year-old Caucasian female-to-male transgender individual (hereafter referred to as a male) suffering from severe non-treatment-resistant major depressive disorder with suicidal ideation. Due to fatigue caused by his mental health issues, he has not completed his transition (i.e., mastectomy at 20 years old, but no hormone therapy).

His family history was the following: major depressive disorder (MDD) in his father; autism spectrum disorders (ASD) in his father’s family members; generalized anxiety disorder in his mother; attention deficit hyperactivity disorder (ADHD); and ASD in his brother.

He has a medical history of multiple psychiatric problems. First of all, he developed in childhood post-traumatic stress disorder (PTSD) and dissociative identity disorder (DID) related to trauma (i.e., physical traumas and sexual abuse). Then, at 12 years old, he started suffering from unipolar MDD. Until the first time we met, he had six major depressive episodes, with one hospitalization in a psychiatric hospital at 17 years old. On four occasions, he attempted suicide by voluntary drug intoxication. In adolescence, around the age of 15, he began to experience significant distress associated with identification with a gender different (male) from that corresponding to the sex designated at birth (female). He was then diagnosed at the age of 19 with gender dysphoria, which motivated him to begin transitioning. Later, at 19 years old, he was diagnosed with both ASD and ADHD. Concerning ASD, since his infancy, he has presented persistent deficits in social communication and social interaction, restricted interests, and hyperactivity to sensory input such as noise, light, touching, and smelling. The level of severity was assessed as 1, according to the DSM5. These disorders did not seem to be better explained by an intellectual disability (intellectual development disorder), although an IQ test could not be performed due to the patient's financial difficulties. Concerning ADHD, he did not wish to take methylphenidate treatment. In addition, he reports chronic joint and muscle pain.

The current depressive episode, which had lasted for five months, had been treated with paroxetine, without efficacy (the dosage was not specified). The general practitioner introduced venlafaxine as a second-line treatment at a dose of 37.5 mg per day. The patient did not wish to increase the dose, so the general practitioner referred him to us after four weeks for further therapeutic advice. Indeed, the dosage of venlafaxine was not optimal. For this reason, the patient’s depression was not considered as a treatment-resistant depression. He has not received psychotherapy because he cannot afford it.

DISCO is the first randomized controlled open-label study (NCT03758105), which main purpose is to perform a cost-utility analysis comparing tDCS-treatment as usual (group A) and treatment as usual only (group B) strategies over 12 months in patients suffering from unipolar or bipolar depression after one or two antidepressant failures during the current episode, from a societal perspective [3]. Treatment failure includes both failures to respond to a well-conducted course of treatment in terms of duration and dosage, as well as poor tolerance, resulting in a refusal by the patient to increase doses or to continue taking the treatment.

The patient was offered two options: to increase the venlafaxine dosage to 75 mg and above if necessary or to participate in the DISCO study, which gave him a chance to access tDCS treatment. The patient refused the first option, according to him, because of his poor tolerance to venlafaxine (anxiety). Therefore, the patient was included in the DICSO study and randomly assigned to the tDCS-treatment as the usual arm.

The initial program of tDCS, administered with a Sooma device (Sooma Oy, Helsinki, Finland) using 35-cm^2^ sponges, consisted of 2-mA anodal stimulation of the left dorsolateral prefrontal cortex (DLPFC) and cathodal stimulation of the right orbitofrontal cortex, for 30 minutes per weekday, over a period of three weeks (14 sessions in all, as the patient missed one session).

Efficacy, tolerability, and medication are described in Table 1.

**Table 1:** Efficacy and tolerance of tDCS. CGI: clinical global impression; CRQ: comfort rating questionnaire; EQ5D: EuroQol five dimensions; MADRS: Montgomery-Åsberg depression rating scale; MoCA: Montreal cognitive assessment; tDCS: transcranial direct current stimulation; VAS: visual analogic scale.

	MADRS	MoCA	CGI	EQ5D	CRQ within tDCS	CRQ after tDCS	Medication	Acceptability of tDCS
Day 0	48/60	29/30	6/7	25/100	NA	NA	Venlafaxine 37.5 mg/day (stable during the tDCS treatment)	NA
First tDCS program	2-mA anodal stimulation of the left dorsolateral prefrontal cortex and cathodal stimulation of the right orbitofrontal cortex, for 30 minutes per weekday, over a period of three weeks (14 sessions in all, as the patient missed one session)							
One month after the first tDCS program	23/60 (Response)	30/30	4/7	55/100	9/80	13/80	Venlafaxine 37.5 mg/day + propranolol 40 mg/day in the last four weeks	VAS 9/10 side effects reported: asthenia, headaches, concentration problems, and trauma reactivation
Two months after the first tDCS program	40/60 (Relapse)	NA	5/7	NA	NA	NA	Propranolol 40 mg, venlafaxine 37.5 mg	NA
	Clinical interview: "It's complicated," but he can say that he has regained his motivation since the first tDCS program. He has resumed her artistic practice and enjoys it. Still fluctuating suicidal thoughts and self-harm behavior last month. Describes himself as anxious and more dissociated lately (moments of absence or even amnesia). Occurrence of post-traumatic symptomatology in the last few weeks: flashbacks and nightmares, avoidance behaviors, hypervigilance, and sleep disorders.							
Second tDCS program seven weeks after M2	30/60	2-mA anodal stimulation of the left dorsolateral prefrontal cortex and cathodal stimulation of the right orbitofrontal cortex, for 30 minutes per weekday, over a period of three weeks						
One month after the second tDCS program	15/60 (Response)	NA	NA	NA	NA	NA	NA	NA

## Discussion

Our case report is diagnostically complex. He presents neurodevelopmental disorders (ASD and ADHD), present since birth and diagnosed in adulthood; complex post-traumatic disorders (PTSD and DID), which appeared in the aftermath of repeated traumas in childhood; gender dysphoria, identified in adulthood; and MDD, a complication that appeared later in adolescence.

First, some disorders may have been masked by others and diagnosed late. Adults who have escaped an ADHD diagnosis in childhood will often present to medical providers and therapists with a mood disorder [4].

Secondly, some of these disorders can be considered as comorbidities but also complications, depending on their chronological onset. For example, nearly half of ADHD adults had re-occurring depression [5]. Although the lesbian, gay, bisexual, transgender, and queer/questioning (LGBTQ+) community is often considered to be at higher risk for developing psychiatric disorders due to social stress and an increased risk of psycho-trauma, it should not be considered a key condition for the onset, severity, or resistance of depression. Indeed, depression may be a common complication of all disorders presented by the patient.

In addition, some symptoms may be common to several disorders. For example, MDD and ADHD both share overlapping traits such as focus and concentration impairments, motivation difficulties, self-esteem/self-worth and mood difficulties, appetite and sleep issues, executive functioning difficulties, agitation and irritability, and working memory impairments. As symptoms of inattention, hyperactivity, and impulsivity were evident before the depression, in his childhood history, we were able to diagnose ADHD in our case [4]. In our case, the depressive cognitions (i.e., guilt, worthlessness, hopelessness, and suicidal thoughts), severe anhedonia, and psychomotor retardation were severe enough to confirm the diagnosis of depression. In the same vein, PTSD and depression also share many symptoms [6].

Our clinical case is also complex from a therapeutic point of view. Firstly, it is difficult to treat several psychiatric disorders and their complications. In addition, his therapeutic adherence was fluctuating. The patient accepted some treatments (antidepressants, tDCS), but with a limitation in dose escalation (e.g., venlafaxine), and refused others (e.g., methylphenidate). Tolerance was moderate. Access to psychotherapies was not feasible for our patient, due to financial problems, which limits the possibilities of combination therapy. From a pragmatic point of view, the severe depressive episode with suicidal ideation (MADRS = 48/60) appeared to be the therapeutic priority, especially as the patient agreed with this strategy.

With the very recent exception of electroconvulsive therapy (ECT), neuromodulation in TGD patients is not addressed in the literature [7-11]. Yet, compared to others, lesbian, gay, bisexual, transgender, and queer/questioning (LGBTQ) patients are more likely to have histories of trauma, self-harm, and substance abuse [1]. The clinical response to ECT is apparently just as effective for LGBTQ patients with treatment-resistant mood disorders [10]. The patient we present was initially deemed responsive to tDCS associated with antidepressant medication, as reflected in a >50% reduction in his Montgomery-Åsberg Depression Rating Scale score. Although he did report side effects (asthenia, headache, and impaired attention), they did not prevent him from pursuing the treatment.

In addition to the improvement of the depressive episode, we could expect to observe an improvement of other associated disorders, with the hypothesis of a common physiopathology, and of transnosographic symptoms. However, tDCS seems to have enhanced his post-traumatic symptoms. The exacerbation of PTSD symptoms in conjunction with the improvement in depression is intriguing and suggests the possibility of two distinct psychopathological hypotheses. The first hypothesis is that the severe depressive syndrome presented by the patient is only the negative affect dimension of PTSD and that tDCS with the anode on the left DLPFC may have acted only on this dimension without affecting the other dimensions of PTSD, which may have reactivated them. The second hypothesis is that post-traumatic symptoms were unmasked as his depression abated. This raises the question of proposing a neuromodulation protocol known to be effective in both comorbidities, such as neuromodulation of the right DLPFC D, by repetitive transcranial magnetic stimulation (rTMS) [12] or by cathode on the right DLPFC combined with anode on the left DLPFC [13].

This post-traumatic occurrence may have in fact contributed to the depressive relapse two months after the conclusion of the initial tDCS program. The relapse might also be explained by the low dosage of venlafaxine (37.5 mg). The dosage remained low because the medication was not well tolerated by the patient. These observations point to the potential value of maintenance tDCS [3] alongside the treatment of comorbidities, such as psychotherapy for DID.

Furthermore, though the patient discussed here was not receiving any hormone therapy for his gender transition, it is important that the potential impacts of hormone treatment and sexual reassignment surgery be considered when evaluating tDCS efficacy and tolerability in the TGD population [14,15].

## Conclusions

TGD patients individuals do experience a higher prevalence of mental health disorders than that of the general population or cisgender individuals and experience a greater burden of health disparities compared with their heterosexual/cisgender counterparts. Our case report suggests that combined tDCS and antidepressant therapy have the potential to treat depressive disorders in patients of all gender identities. There is an urgent need to better identify the specific therapeutic needs of TGD patients, in order to offer them adapted psychiatric care, and to reduce inequalities in access to care.
